# *Rehmannia glutinosa* Libosch Extracts Prevent Bone Loss and Architectural Deterioration and Enhance Osteoblastic Bone Formation by Regulating the IGF-1/PI3K/mTOR Pathway in Streptozotocin-Induced Diabetic Rats

**DOI:** 10.3390/ijms20163964

**Published:** 2019-08-15

**Authors:** Wan Gong, Naidan Zhang, Gang Cheng, Quanlong Zhang, Yuqiong He, Yi Shen, Qi Zhang, Bo Zhu, Qiaoyan Zhang, Luping Qin

**Affiliations:** 1 College of Pharmaceutical Sciences, Zhejiang Chinese Medical University, Hangzhou 311402, China; 2 College of Basic Medical Science, Zhejiang Chinese Medical University, Hangzhou 310053, China; 3 School of Pharmacy, Second Military Medical University, Shanghai 200433, China; 4 School of Pharmacy, Fujian University of Traditional Chinese Medicine, Fuzhou 350108, China

**Keywords:** *Rehmannia glutinosa* Libosch, diabetic osteoporosis, osteoblast, BMP pathway, IGF-1/PI3K/mTOR signaling pathways

## Abstract

Rehmanniae Radix Praeparata (RR, named as Shudihuang in traditional Chinese medicine), the steamed roots of *Rehmannia glutinosa* Libosch (Scrophulariaceae), has been demonstrated to have anti-diabetic and anti-osteoporotic activities. This study aimed to explore the protective effect and underlying mechanism of RR on diabetes-induced bone loss. It was found that RR regulated the alkaline phosphatase activity and osteocalcin level, enhanced bone mineral density, and improved the bone microarchitecture in diabetic rats. The catalpol (CAT), acteoside (ACT), and echinacoside (ECH) from RR increased the proliferation and differentiation of osteoblastic MC3T3-E1 cells injured by high glucose and promoted the production of IGF-1 and expression of related proteins in BMP and IGF-1/PI3K/mammalian target of rapamycin complex 1 (mTOR) signaling pathways. The verifying tests of inhibitors of BMP pathway (noggin) and IGF-1/PI3K/mTOR pathway (picropodophyllin) and molecular docking of IGF-1R further indicated that CAT, ACT, and ECH extracted from RR enhanced bone formation by regulating IGF-1/PI3K/mTOR signaling pathways. These findings suggest that RR may prove to be a promising candidate drug for the prevention and treatment of diabetes-induced osteoporosis.

## 1. Introduction

Diabetes mellitus (DM) and osteoporosis are common diseases with increasing prevalence in the aging population [1]. DM is a hyperglycemic metabolic syndrome that may cause an imbalance of bone metabolism, finally leading to bone loss and osteoporosis [2]. Bone loss or osteoporosis induced by DM, known as diabetic osteoporosis (DOP), is characterized by poor bone healing and regeneration, and increased risk of bone fractures [3]. Accumulating evidence has shown that the risk of osteoporotic fractures is significantly increased in both type 1 (T1)DM and type 2 (T2)DM patients [4]. A meta-analysis also showed that the hip fracture risk in T1DM and T2DM patients was 6.3-fold and 1.7-fold that in non-diabetes patients, respectively [5]. The treatment costs of bone fractures in diabetic patients are increased due to prolonged wound healing and other complications. With the sharp increase in diabetic populations, diabetic osteoporosis, which often causes more pains and increased risk of fractures for DM patients, has become a clinical challenge that needs to be addressed. It is therefore necessary to develop an effective strategy for the prevention and treatment of bone loss and osteoporosis induced by DM.

There is growing evidence supporting that DM may result in disturbance of bone metabolism [6]. Several studies [7,8] demonstrated that DM induced bone loss and reduced bone mechanical properties with a decreased osteoblastic bone formation rate. Insulin-like growth factors (IGFs), which are known to be involved in the regulation of blood glucose and play an important role in bone remodeling, are produced and stored in the bone matrix [9]. It was reported [10,11] that IGF-1 could stimulate osteoblast proliferation and differentiation by activating the expression of the mammalian target of rapamycin complex 1 (mTOR) via PI3K-Akt pathway, and therefore the IGF-1/PI3K/mTOR pathway is believed to be involved in the regulation of osteoblastic bone formation in the pathophysiology of diabetic osteoporosis.

Rehmanniae Radix praeparata (RR), also known as Shudihuang in Chinese, is the steamed root of *Rehmannia glutinosa*, which is deemed to nourish yin and invigorate the kidney in Traditional Chinese Medicine (TCM) and has long been used for the treatment of DM, osteoporosis, and cardiovascular disease in China [12]. Phytochemical and pharmacological investigations [13] show that RR contains various compounds including catalpol (CAT), dihydrocatalpol, acteoside (ACT), and echinacoside (ECH), which possess a variety of bioactivities such as anti-diabetes, anti-osteoporosis, and protective effects against hematological and gynecological diseases. The anti-diabetic activities of RR have been evidenced in streptozotocin (STZ)-induced diabetic mice, including effectively ameliorating hyperglycemia, hyperlipemia, vascular inflammation, and oxidative stress [14]. CAT extracted from RR has been confirmed as an active anti-diabetic constituent by ameliorating structural impairment of the pancreas, restoring redox balance, improving renal functions, and pathological changes in the kidneys through up-regulation of IGF-1 expression and IGF-1R phosphorylation in diabetic mice induced by STZ combined with high-fat and high-sugar feed [15]. RR was also found to alleviate the decrease in trabecular bone mineral density (BMD) and cortical bone thickness in OVX-induced osteoporotic rats, increase the proliferation, alkaline phosphatase (ALP) activity and expression of osteoprotegerin in osteoblasts, and decrease the number of tartrate-resistant acid phosphatase (TRAP) positive multinucleated cells and the resorption areas of osteoclasts [16,17]. CAT has also been reported to promote osteoblast proliferation and differentiation in MC3T3-E1 cells [18]. ACT extracted from RR has been found to reduce OVX-induced bone loss and inflammatory cytokine production, inhibit osteoclast differentiation and maturation by suppressing RANKL-induced activation of MAPK and transcription factors such as NF-κB, c-Fos, and NFATc1 [19]. However, the effects of RR and its active constituents on DM-induced bone loss remain unclear. The aim of the present study was to observe the protective effects of RR against bone loss in a diabetic rat model induced by STZ combined with high fat and high glucose diet, clarify the effects of active components in RR on MC3T3-E1 osteoblasts damaged by high glucose, and explore the underlying action mechanisms of these active constituents in RR in regulating bone formation through the IGF-1/PI3K/mTOR pathways.

## 2. Results

### 2.1. Effects of RR on the Body Weight and Levels of Random Blood Glucose in Diabetics Rats

**Figure 1:**
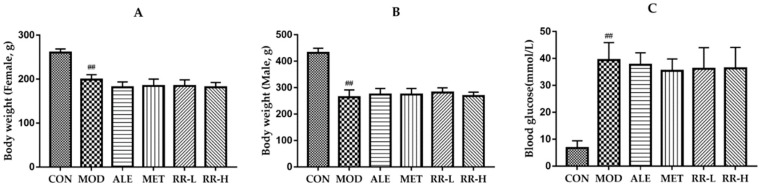
Effects of Rehmanniae Radix Praeparata (RR) on body weight and random blood glucose levels in diabetic rats after 8-week oral administration. (**A**) body weight of female rats; (**B**) body weight of male rats; (**C**) random blood glucose levels. All data are expressed as mean ± SD (*n* = 5 for body weight; *n* = 10 for random blood glucose level). ^##^ *p* < 0.01 as compared to control group.

The body weight and levels of random blood glucose of the rats at the end of the experiment are shown in Figure 1. The body weight in the model groups was significantly lower than that in the CON group, while there was no significant difference in body weight between the model groups and alendronate sodium treatment (ALE), metformin treatment (MET), and RR treatment groups. In comparison with the rats in the control group, the rats in the model groups also displayed a higher level of random blood glucose. ALE treatment did not produce any effect on the level of random blood glucose in diabetic rats. RR and MET caused a slight decrease in random blood glucose level in diabetic rats, but these differences were not significant, compared with the rats in the model groups.

### 2.2. RR Regulates Biochemical Parameters Related to Bone Formation in Diabetic Rats

**Figure 2:**
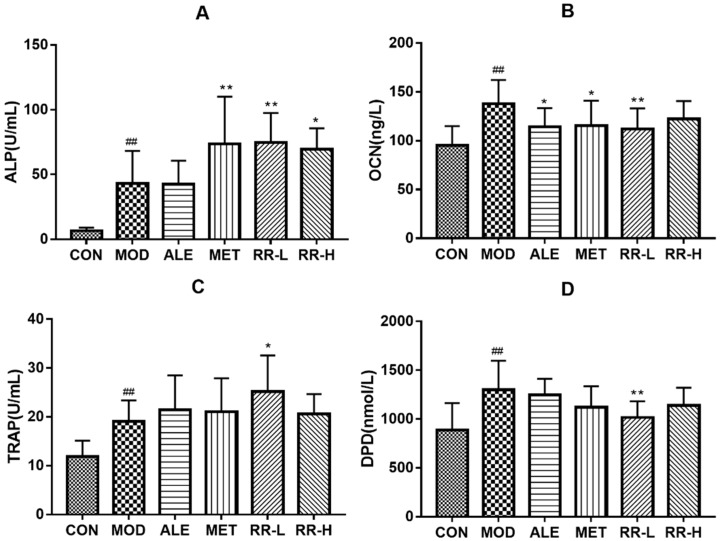
Effects of RR on biochemical parameters related to bone metabolism in diabetic rats after 8-week oral administration. (**A**) Serum alkaline phosphatase (ALP) activity. (**B**) Serum osteocalcin (OCN) level. (**C**) Serum tartrate-resistant acid phosphatase (TRAP) activity. (**D**) Urine deoxypyridinoline (DPD) level. All data are expressed as the mean ± SD (*n* = 10). ^##^ *p* < 0.01 as compared with the control group, and * *p* < 0.05 and ** *p* < 0.01 as compared with the model group.

The biochemical markers of bone metabolism are shown in Figure 2. Serum bone-specific ALP activity and serum osteocalcin (OCN) level, two important indicators of bone formation, were significantly increased in the diabetic rats, compared with those in the normal control rats. ALE decreased the OCN level but had no significant effect on the ALP activity in diabetic rats. MET increased the ALP activity in diabetic rats, while RR increased the ALP activity and simultaneously decreased the OCN level in the serum of diabetic rats. Serum TRAP activity and urine deoxypyridinoline (DPD) level, two critical markers of bone resorption, were significantly enhanced in diabetic rats, compared with those in the normal control rats. ALE and MET did not produce any effect on TRAP and DPD levels in diabetic rats, and RR decreased the urine DPD level in diabetic rats at a dose of 1 g/kg. These results indicate that RR was mainly involved in the regulation of osteoblastic bone formation, but not osteoclastic bone resorption.

### 2.3. RR Enhances Bone Mineral Density and Improves the Bone Microarchitecture in Diabetic Rats

**Figure 3:**
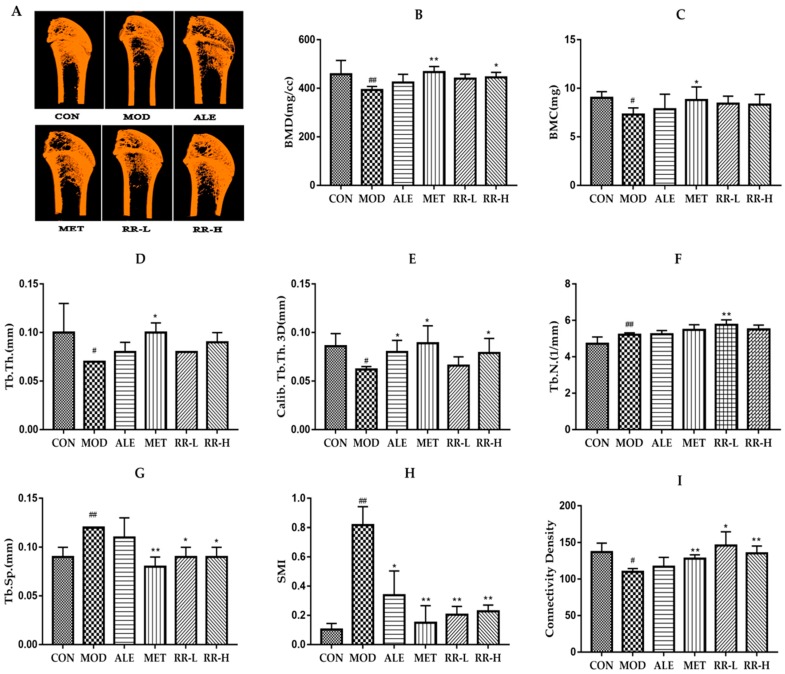
Effects of RR on bone mineral density and histological morphometric alteration of the femurs in diabetic rats. (**A**) Representative 3D micro-CT images of the distal femoral trabecular bone micro-architecture; (**B**) bone mineral density (BMD); (**C**) bone mineral content (BMC); (**D**) trabecular thickness (Tb.Th); (**E**) 3D calibration of trabecular thickness (calib.Tb.Th.3D); (**F**) trabecular number (Tb.N); (**G**) trabecular separation (Tb.Sp); (**H**) structure model index (SMI); and (**I**) connectivity density. All data are expressed as the mean ± SD (*n* = 5). ^#^ *p* < 0.05 and ^##^ *p* < 0.01 as compared with the control group, and * *p* < 0.05 and ** *p* < 0.01 as compared with the model group.

Micro CT analyses were used to evaluate the alteration of the trabecular bone microarchitecture in diabetic rats. As shown in Figure 3, the representative micro-CT images revealed an obvious decrease in trabecular bone mass and deterioration of the cancellous bone microarchitecture in diabetic rats compared with the normal control rats. ALE, MET, and RR treatment for 8 weeks showed an obvious preventive effect against trabecular bone architectural deterioration in diabetic rats. In addition, bone histological morphometric analysis demonstrated that BMD, bone mineral content (BMC), trabecular thickness (Tb.Th), 3D calibration of trabecular thickness (Calib.Tb.Th.3D), and connectivity density were significantly reduced, and trabecular separation (Tb.Sp) and structure model index (SMI) were significantly increased in the femurs of diabetic rats. ALE treatment could improve the above parameters, but only exert significant effects on Calib.Tb.Th.3D and SMI in the femurs of diabetic rats. MET could improve bone histological morphometric parameters except for trabecular number (Tb.N) in diabetic rats. RR treatment significantly enhanced BMD, Calib.Tb.Th.3D, Tb.N, and connectivity density, and reduced Tb.Sp and SMI in the femurs of diabetic rats, indicating that RR could prevent bone loss and deterioration of the microarchitecture in diabetic rats.

### 2.4. CAT, ACT, and ECH Extracted from RR Enhance the Proliferation and Differentiation of Osteoblastic MC3T3-E1 Cells Damaged by High Glucose

**Figure 4:**
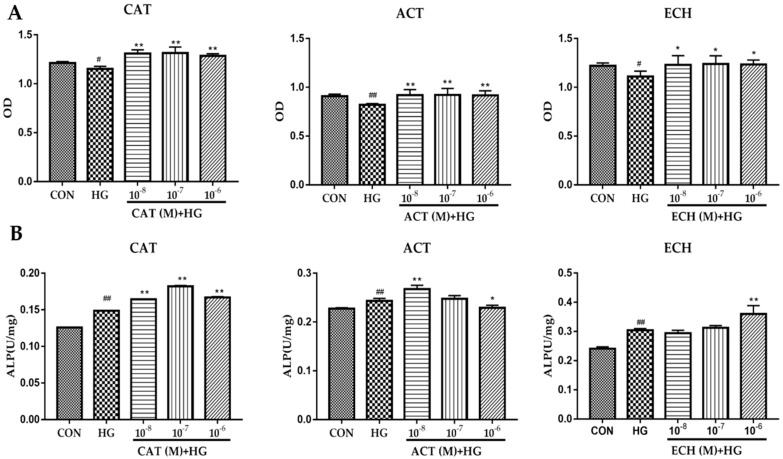
Effects of catalpol (CAT), acteoside (ACT), and echinacoside (ECH) against high-glucose induced injury to osteoblastic MC3T3-E1 cells. The osteoblastic MC3T3-E1 cells were treated with CAT, ACT, and ECH for 48 h, and then injured with 200 mM glucose for 48 h. (**A**) Cell viability was assayed with a CCK-8 kit (*n* = 5); (**B**) ALP activity was determined by using an ALP activity assay kit (*n* = 3). All data are expressed as the mean ± SD. ^#^ *p* < 0.05 and ^##^ *p* < 0.01 as compared with the normal control group, and * *p* < 0.05 and ** *p* < 0.01 as compared with the high-glucose treatment group.

Knowing that RR could prevent bone loss in diabetic rats through regulating the osteoblastic bone formation, the effects of CAT, ACT, and ECH extracted from RR against high-glucose induced injury to osteoblastic MC3T3-E1 cells were evaluated. As shown in Figure 4, CAT, ACT, and ECH could significantly increase the proliferation and ALP activity in high glucose-injured osteoclastic MC3T3-E1 cells, indicating that CAT, ACT, and ECH could improve the proliferation and differentiation of osteoblast.

### 2.5. CAT, ACT, and ECH Modulate the BMP2 Pathway in Osteoblastic MC3T3-E1 Cells

**Figure 5:**
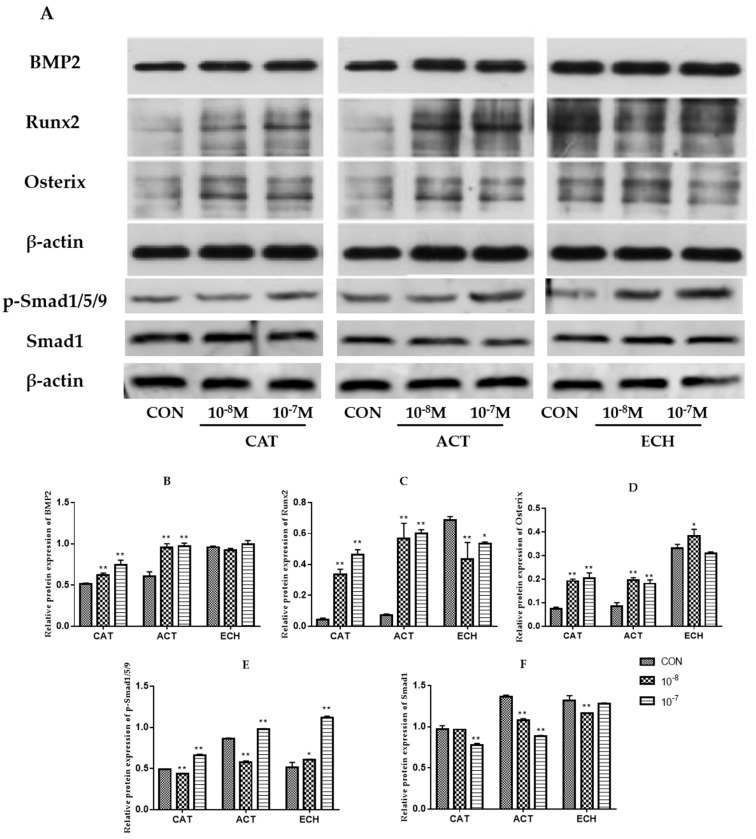
Effects of CAT, ACT, and ECH on the expression of BMP2, Runx2, Osterix, p-Smad1/5/9 and Smad1 in osteoblastic MC3T3-E1 cells (**A**–**F**). β-actin served as a protein loading control. The expression of key proteins in the BMP pathway was analyzed by Western-blot method. All data are expressed as the mean ± SD (*n* = 3), * *p* < 0.05 and ** *p* < 0.01 as compared with the control group.

BMP2 promotes osteoblastogenesis through upregulating the expression of Runx2 and Osterix, thus stimulating bone formation [20]. As shown in Figure 5 and Figures S1 and S2, CAT and ACT increased the expression of BMP2, Runx2, Osterix, and p-Smad1/5/9, and ECH exhibited promoting effects on Osterix and p-Smad1/5/9 in MC3T3-E1 cells, indicating that CAT, ACT, and ECH increased bone formation through regulating the BMP pathway.

### 2.6. CAT, ACT, and ECH are Involved in Regulation of IGF-1/PI3K/mTOR in MC3T3-E1 Cells

**Figure 6:**
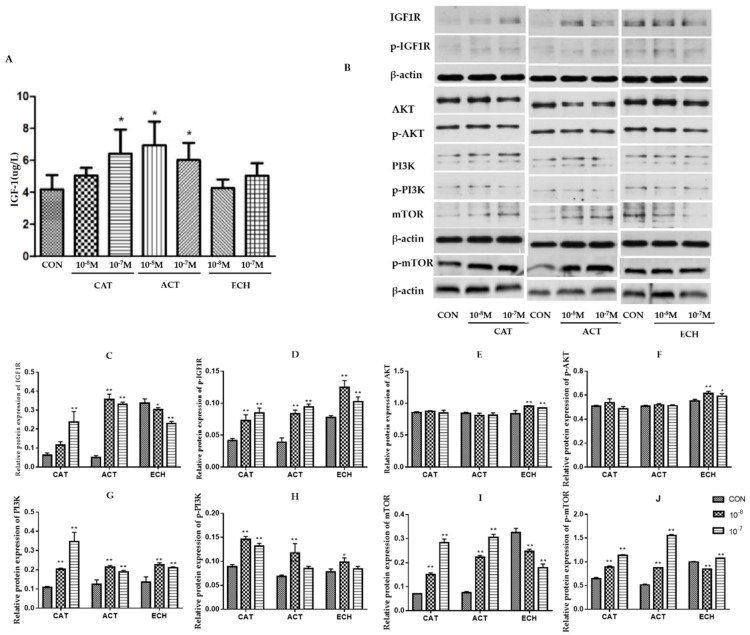
CAT, ACT, and ECH are involved in the regulation of IGF-1/PI3K/mTOR pathways in osteoblastic MC3T3-E1 cells. (**A**) IGF-1 level secreted in MC3T3-E1 cells (*n* = 5); (**B**–**J**) the expression of key proteins in IGF-1/PI3K/mTOR pathways in MC3T3-E1 cells was analyzed by Western-blot method (*n* = 3). All data are expressed as the mean ± SD, * *p* < 0.05 and ** *p* < 0.01 as compared with the control group.

IGF-1 plays a vital role in osteoblastic bone formation through the IGF-1/PI3K/mTOR pathway. As shown in Figure 6 and Figures S3 and S4, CAT and ACT significantly stimulated the production of IGF-1 in MC3T3-E1 cells, increased the expression and phosphorylation of IGF-1R, and enhanced the phosphorylation of PI3K and mTOR, but had no effect on the expression of AKT and p-AKT. Meanwhile, ECH decreased the expression of IGF-1R and mTOR, but increased the phosphorylation of IGF-1R. ECH can also enhance the expression and phosphorylation of PI3K and AKT in MC3T3-E1 cells. 

### 2.7. Noggin (NOG) and Picropodophyllin (PPP) Counteract the Effects of CAT, ACT, and ECH in MC3T3-E1 Cells

**Figure 7:**
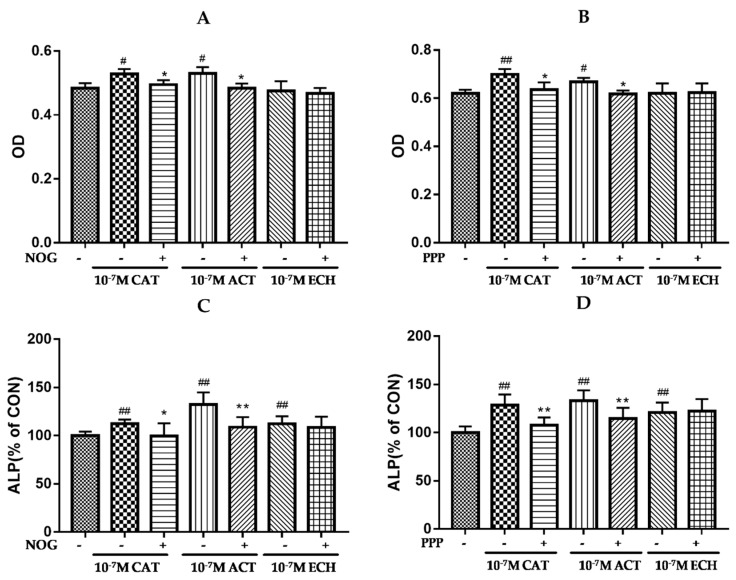
Noggin (NOG) and picropodophyllin (PPP) counteract the effects of CAT, ACT, and ECH on MC3T3-E1 cells. MC3T3-E1 cells were treated with CAT, ACT, and ECH at a concentration of 10^−7^ M, or combined with NOG (200 ng/mL) or PPP (2 × 10^−7^ M) for 48 h. (**A**,**B**) Cell viability was assayed using a CCK-8 kit (*n* = 6); (**C**,**D**) ALP activity was determined by using an ALP activity assay kit (*n* = 3). All data are expressed as the mean ± SD. ^##^ *p* < 0.01 and ^#^ *p* < 0.05 as compared with the control group; ** *p* < 0.01 and * *p* < 0.05 as compared with the CAT, ACT, or ECH treatment group.

Noggin and PPP are inhibitors of the BMP and IGF-1/PI3K/mTOR pathways, respectively. As shown in Figure 7, CAT and ACT increased the proliferation and ALP activity of osteoblastic MC3T3-E1 cells, while noggin and PPP attenuated the effects of CAT and ACT in osteoblasts. However, ECH increased ALP activity and exhibited no significant effect on osteoblast proliferation. These results indicate that CAT and ACT extracted from RR could promote osteoblastic bone formation by regulating BMP and IGF-1/PI3K/mTOR pathways of osteoblasts. 

### 2.8. Molecular Docking Studies

**Figure 8:**
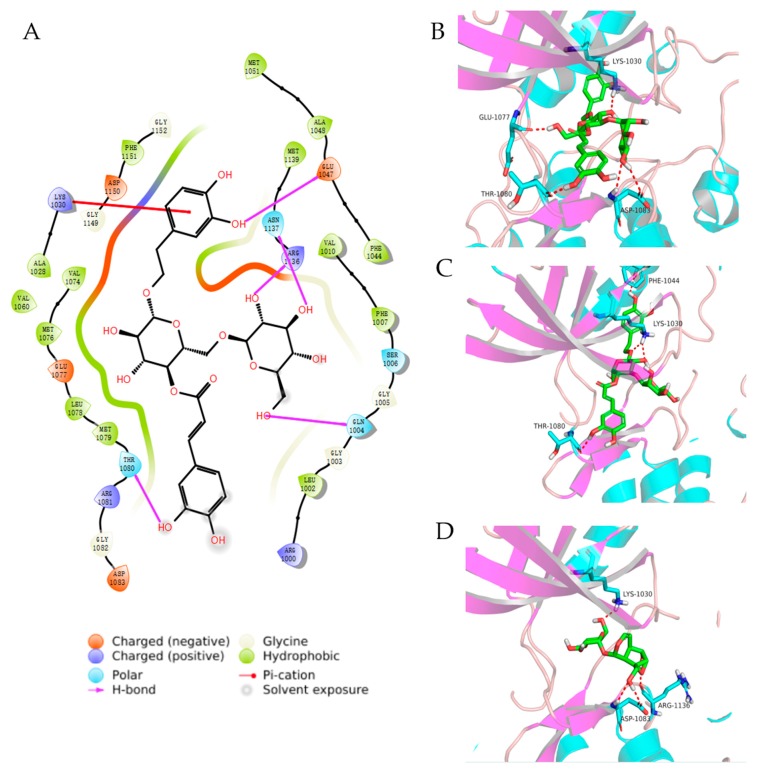
Predicted binding mode of ECH_rm_both_sugar (**A**,**B**), ACT (**C**) and CAT (**D**) with IGF-1R.

To substantiate on the above findings, we performed molecular docking analysis on IGF-1R crystal structures to predict whether CAT, ACT, and ECH, as well as their deglycosylation derivates, interacts with IGF-1R proteins (PDB ID: 5HZN) [21] by adopting the glide module from the Schrodinger suite [22]. The docking estimation was performed by the docking score. The lower docking score values imply more favorable binding affinities between the protein and the ligand. The docking score for compounds ECH_rm_both_sugar (two sugar fragments have been removed from ECH), ACT, and CAT were −10.8, −10.2 and, −6.3 respectively; and the ligand from the crystal complex was −12.9. Thus, the docking energies were consistent with the Western blot results, which may further explain for regulatory effects of CAT, ACT, and ECH on IGF-1/PI3K/mTOR. The binding mode of compound ECH_rm_both_sugar, ACT, and CAT are provided in Figure 8. The whole molecule of ECH_rm_both_sugar fits well in the active site of IGF-1R surrounded by ME1139, VAL1010, PHE1007, MET1079, LEU1078, MET1076, and VAL1076, etc, and four hydrogen bonds were formed with ASP1083, THR1080, GLU1077, and LYS1030. ACT also fits well in the same binding pocket of IGF-1R and has a very similar bonding configuration as ECH_rm_both_sugar, and makes three hydrogen contacts in this pocket with THR1080, PHE-1044, and LYS-1030. CAT also makes three hydrogen contacts in this pocket with LYS-1030, ARG-1136, and ASP-1083. 

## 3. Discussion

The present study demonstrated for the first time that RR treatment for 8 weeks significantly prevented DM-induced deterioration of the bone microarchitecture through inhibiting the reduction of bone formation, and that CAT, ACT, and ECH extracted from RR increased the bone formation of osteoblasts damaged by high glucose. They were also found to be involved in the regulation of the IGF-1/PI3K/mTOR pathway in osteoblastic MC3T3-E1 cells. 

Micro-CT analysis of the present study demonstrated that RR exerted a bone protective effect in STZ-induced diabetic rats. RR treatment significantly inhibited further deterioration of the femoral trabecular bone microstructure in diabetic rats, as evidenced by enhanced BMD, Calib.Tb.Th.3D, Tb.N, and connectivity density, and reduced Tb.Sp and SMI. The observed cancellous bone deterioration in STZ-treated diabetic rats is consistent with the finding from other investigators and similar to the cancellous bone status in clinical diabetic patients [23]. Several previous studies [16,17] suggested that RR treatment increased bone mass in both normal and osteoporotic animals induced by ovariectomy. The findings in the present study may enrich the understanding of the therapeutic efficacy of RR on bone tissues and support the conclusion that RR treatment prevents trabecular bone deterioration in diabetes.

The serum biochemical results in our study showed that bone formation was increased in STZ-treated diabetic rats, as represented by higher serum OCN secretion and bone-specific ALP activity, and enhanced bone resorption as evidenced by higher TRAP activity and DPD level in diabetic rats, indicating that STZ and high-fat diet caused disturbance of bone metabolism. RR treatment increased the serum level of bone-specific ALP activity and reduced the OCN level, but not the TRAP activity and DPD level, showing that RR is involved in the regulation of bone formation, but not the bone resorption in diabetic rats. The anabolic effects of RR on bone formation were also further confirmed through observing the effects of CAT, ACT, and ECH extracted from RR on osteoblastic MC3T3-E1 cells injured by high glucose. 

The serum ALP and OCN levels as biochemical bone turnover markers have been widely studied in diabetes patients. It seems that the markers may be lower in diabetes patients, but with great heterogeneity between investigations [24]. Kemink et al. reported that there was no difference in the mean serum ALP and OCN levels between diabetic patients and the normal controls [25]. Xie et al. found a significantly decreased serum OCN levels and ALP activity in diabetic rats induced by STZ plus high-fat diet [26]. Choi et al. reported that serum ALP levels in rats induced by STZ after 42 days were significantly increased [27], and Xiao et al. found that serum ALP levels in animals consumed high-carbohydrate, high-fat diet and administered two intraperitoneal injections of STZ at 4 (25 mg/kg) and 8 (40 mg/kg) weeks after diet initiation showed a significant surge at 8 weeks, and followed by a decline at 16 weeks, while the levels of serum OCN consistently decreased [28]. The increase of ALP and OCN in serum may be related to secretion and release of IGF-1 of osteoblast. Some studies showed that IGF-1, a hormone similar in molecular structure to insulin and highly present and secreted in osteoblasts, significantly decreased in diabetes patients and animals, which could lead to the acceleration of bone resorption [29]. Then IGF-1 released from the bone matrix resorptive sites induced the ALP activity in osteoblasts, thereby increasing the mineralization of bone matrix [30]. Moreover, with the elevation of bone resorption, OCN was also released from bone matrix into blood, leading to a higher level of OCN in the blood of diabetic osteoporotic patients and animals [4]. The increase of ALP and OCN enhanced the accumulation and mineralization of bone minerals; on the other hand, hyperglycemia inhibited the synthesis of bone matrix proteins such as collagen and osteopontin. This imbalance of bone mineral and matrix proteins resulted in an increased bone mineral density and decreased bone flexibility, and finally leading to higher fracture risk [3]. In our study, the rats were fed with a high-fat diet for 4 weeks, and then treated with STZ to induce diabetes, the ALP activity and OCN levels in serum were increased in diabetic rats after 8 weeks of injection of STZ, these results are identical with Choi and Xiao’s research.

The change of ALP activity in osteoblast injured by high glucose is also contradictory in currently reported literature. Treatment of MC3T3-E1 cells with 25.5 mM glucose for 14 days led to a significant decrease in osteoblastic proteins, such as collagen I, Runx2, and ALP activity [31], and the treatment with 33 mM glucose decreased the level of ALP in osteoblastic MC3T3-E1 cells at the time of treatment of 3, 9, 15, and 25 days [32]. However, Botolin and McCabe [33] reported that treatment of osteoblastic MC3T3-E1 cells with 30 mM glucose significantly increased the ALP activity at 7, 14, 21, and 29 days, and acute hyperglycemia for a 48 h period also caused an increase in ALP activity. The increase of ALP activity may be related to cell culture conditions of high glucose. Under the condition of hyperglycemia, the osmotic pressure is increased; the pH is decreased in the medium by increasing extracellular lactic acid production or another byproduct of increased glucose metabolism [33]. The osteoblast undergoes a volume change and shrinks to adapt to extracellular hyperosmolality, and responds to decreased extracellular pH by reducing mineralization and gene expression [34]. However, the alkaline phosphatase expression was markedly induced by hyperglycemia at 48 h treatment and the longer treatment [33]. The elevation of ALP activity may be owing to its developing adaptive measures to compensate for the hyperosmotic stress and lower extracellular pH in the culture medium. Owing to the elevated ALP activity, the BMD may be increased in the diabetic osteoporotic patient, but the fragility of bone is also enhanced, leading to a higher risk of bone fracture in the diabetic patient [3]. This indicated that the prevention and treatment for diabetic osteoporosis should improve both bone mineral formation and collagen protein accumulation. In the present study, treatment of osteoblastic MC3T3-E1 cells with 200 mM glucose for 48 h significantly decreased the proliferation and increased the ALP activity, and CAT, ACT, and ECH from RR could enhance the proliferation and ALP activity of osteoblast, indicating that CAT, ACT, and ECH from RR could modulate the disturbance of osteoblastic bone formation under high glucose conditions.

The BMP signaling pathway is known to play vital roles in osteoblast differentiation [35]. BMPs enhance osteoblastogenesis through the Smad and MAPK pathways by increasing the expression of Runx2 and Osterix and promoting bone formation [36,37]. Runx2 is a master regulator of osteoblastogenesis through multiple signaling pathways. Osterix (Osx), also known as Sp7, is a zinc-finger transcription factor in osteoblasts and indispensable for bone formation [36]. Runx2 and Osterix inhibit adipocyte differentiation [38] and stimulate bone formation by regulating the expression levels of specific osteoblast differentiation markers of ALP, OPN, bone sialoprotein, and OCN [39,40,41]. BMP2 is known to regulate the expression and functions of Runx2 through Smad signaling [42]. The results of the present study demonstrated that CAT and ACT could increase the expression of BMP2, Runx2, Osterix, and p-Smad1/5/9 markedly, and ECH exhibited promoting effects on Osterix and p-Smad1/5/9 in MC3T3-E1 cells, suggesting that one of the potential mechanisms of CAT, ACT, and ECH in promoting bone formation is concerned with the regulation of the BMP signaling pathway.

**Figure 9:**
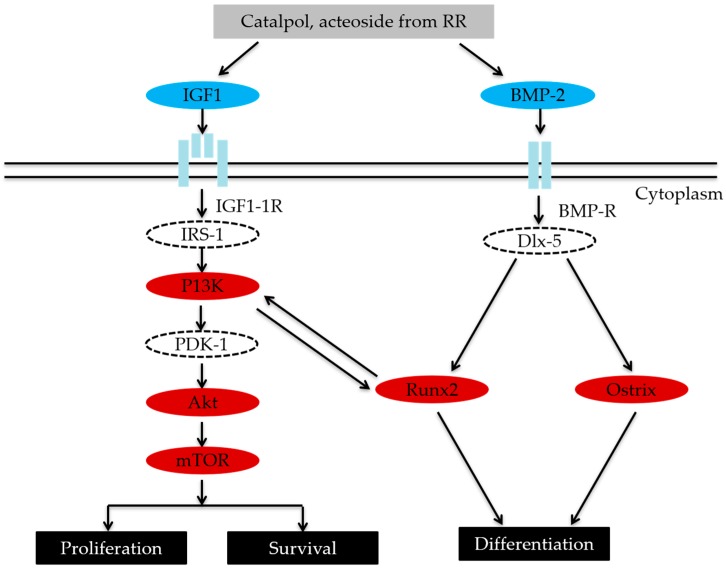
CAT and ACT from RR increased osteoblastic bone formation via BMP and IGF-I signal transduction pathways.

IGF-1 can regulate bone formation and remodeling by affecting the proliferation and survival of osteoblasts [43,44]. The bone anabolic effect of IGF-1 was achieved via PI3K/AKT/mTOR pathways [10]. It is worthy to point out that Runx2 and PI3K/Akt/mTOR pathway are mutually dependent on each other in the regulation of osteoblast differentiation. Runx2 up-regulated the protein levels of PI3K subunits and Akt, whereas PI3K/Akt/mTOR pathway greatly strengthened the DNA binding of Runx2 and Runx2-dependent transcription. This positive feedback loop further promotes Runx2 activity in osteoblast differentiation and their migration [45]. The present investigation found that CAT and ACT from RR not only enhanced the secretion of IGF-1 but increased the protein expression of p-IGF-1R, p-PI3K, and p-mTOR in osteoblastic MC3T3-E1 cells. Knowing that CAT and ACT from RR were involved in the modulation of both BMP and Runx2, and PI3K/Akt/mTOR pathways of osteoblasts, we speculated that CAT and ACT from RR increased bone formation probably through enhancing the secretion of IGF-1 and regulating the function of osteoblasts through the BMP and PI3K/Akt/mTOR pathways (Figure 9).

In addition, molecular docking, the widely used method in the fields of drug design and virtual screening, is performed in this study. The results showed ECH, ACT, and CAT could bond properly within the binding pocket of IGF-1R, which further indicated that one of the targets of ECH, ACT, and CAT may be IGF-1R. CAT is more weakly combined with the binding pocket of IGF-1R than that of ACT and ECH, but could significantly regulate the expression and phosphorylation of IGF-1R, this may be because CAT could also regulate the expression of upstream proteins of PI3K/Akt/mTOR signaling pathways. ECH could strongly bind with the pocket of IGF-1R, but weakly regulated the protein expression of PI3K/Akt/mTOR pathways, this inconsistency needs further investigated.

RR contains a variety of chemical constituents, such as iridoid glycosides, phenylethanoid glycosides, and polysaccharides. In this study, it was found that CAT (iridoid glycosides), ACT, and ECH (phenylethanoid glycosides) had strong interactions with IGF-1R, so IGF-1R may be the target of RR, but it needs further verification. This study showed that CAT, ACT, and ECH could increase the formation and differentiation of osteoblasts by regulating the PI3K/Akt/mTOR pathway and BMP pathway. Moreover, it has been reported that RR could also regulate the formation and differentiation of osteoblasts through the Wnt/beta-catenin pathway [46,47]. Therefore, RR may regulate bone metabolism through multiple targets and pathways to reduce bone loss.

## 4. Materials and Methods

### 4.1. Materials

RR was purchased from Shanghai Dekang Pharmaceutical Co. Ltd. (Shanghai, China), and identified as the root of *Rehmannia glutinosa* Libosch by Professor Qiao-Yan Zhang. The voucher specimen (No. 2015007) was deposited in the herbarium of the Department of Pharmacognosy of the Second Military Medical University (Shanghai, China). RR was extracted with 8-fold water x 3. Then, the mixture was filtered and the filtrate was evaporated under reduced pressure. CAT and ACT were obtained from Yuanye Biological Technology Co. Ltd. (Shanghai, China). ECH was obtained from Chenguang Biological Technology Co. Ltd. (Baoji, China). Metformin hydrochloride tablets were purchased from Shiguibao Pharmaceutical Co. Ltd. (Shanghai, China). Alendronate sodium tablets were obtained from MSD pharmaceutical Co. Ltd. (Hangzhou, China). Streptozotocin was obtained from Sigma-Aldrich (St Louis, MO, USA). Picropodophyllin was obtained from Selleck (Shanghai, China). DPD, OCN, and IGF-1 ELISA kits were purchased from Xinyu Biological Technology Co. Ltd. (Shanghai, China). ALP and TRAP kits were purchased from Jiancheng Bioengineering Institute (Nanjing, China). The BCA Kit was purchased from Beyotime Institute of Biotechnology (Shanghai, China).

### 4.2. Animal Experimental Protocols

Male and female Wistar rats aged 8 weeks (Slac Laboratory Animal Co. Ltd., Shanghai, China) were housed under controlled room temperature (22 ± 3 °C) and humidity (40%–70%) with a 12-h light/dark cycle with free access to water and food. All animal protocols were approved by the Experimental Animal Ethics Committee of the Second Military Medical University (2016LY0916, 16 September 2016).

**Figure 10:**
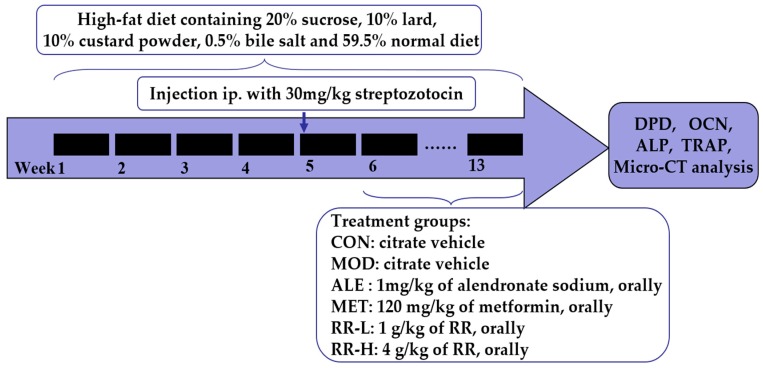
Animal experimental procedures. Wistar rats were fed with a high-fat diet for 4 weeks, and then intraperitoneally injected with streptozotocin to induce hyperglycemic syndrome, and then treated with RR, alendronate sodium and metformin for an additional 8 weeks.

Rats, except for those in control group, were fed with a high-fat diet containing 20% sucrose, 10% lard, 10% custard powder, 0.5% bile salt, and 59.5% normal diet in the former 4-week experiment period, and then intraperitoneally (i.p.) injected with streptozotocin. Rats in the control group were injected with 2 mL/kg 0.1 mM vehicle citrate buffer (pH 4.4). After 7-day streptozotocin injections, venous blood glucose levels of the rats were measured using a glucometer (MAJOR II Blood Glucose Monitoring System, Taiwan), and rats with a glucose level higher than 16.7 mmol/L were considered diabetic and chosen for further study. Then, animals were supplied with the vehicle or drug for an additional 8 weeks (Figure 10). Sixty rats were equally randomized to six groups: (1) A control (CON) group, where normal rats were orally administered with the citrate vehicle; (2) a diabetic model (MOD) group, where diabetic rats were orally administered with the citrate vehicle; (3) an alendronate sodium treatment (ALE) group, where diabetic rats were orally administered with 1 mg/kg alendronate sodium; (4) a metformin treatment (MET) group, where diabetic rats were orally administered with 120 mg/kg metformin; (5) a RR-L treatment (RR-L) group, where diabetic rats were orally administered with 1 g/kg of RR; and (6) a RR-H treatment (RR-H) group, where diabetic rats were orally administered with 4 g/kg of RR.

At the end of the experiment, all rats were placed in metabolic cages and fasted to collect 12-h urine. Then all rats were anesthetized using 3 mL/kg 10% (*w*/*v*) chloral hydrate via i.p. injection. The blood was sampled via the abdominal aorta, and centrifuged at 3000 rpm for 30 min to collect serum; the serum was stored at −80 °C until analysis. Then, rats were sacrificed, and the right femurs were removed and stored in 10% formaldehyde for micro-CT analysis.

### 4.3. Assay for Biochemical Parameters Related to Bone Metabolism

Urine level of DPD and serum level of OCN were determined using ELISA kits. Serum bone-specific ALP and TRAP activities were quantified by corresponding assay kits according to the manufacturer’s instructions.

### 4.4. Micro-CT Analysis

The trabecular bone mass and microarchitecture of the right femurs were detected with a micro-computed tomography (micro-CT) scanner (GE explore Locus SP, Fairfield, CT, USA) and analyzed by micro-view bone analysis software (GE healthcare version 2.1.2, Fairfield, CT, USA). The proximal segment of the femur was dissected and measured with micro-CT. The 3D microarchitecture was re-established with software, and the region of interest (ROI) was selected for analysis. The micro-CT quantitative parameters were obtained, including BMD, BMC, Tb.Th, Tb.N, Tb.Sp, Calib.Tb.Th.3D, connectivity density, and SMI.

### 4.5. Cell Culture

MC3T3-E1 osteoblast-like cells, established from C57BL/6 mouse calvaria, were purchased from the Cell Bank of the Chinese Academy of Sciences (Shanghai, China). The cells were incubated in α-MEM medium (Gibco, Grand Island, NY, USA) supplemented with 10% fetal bovine serum (FBS, Gibco, Grand Island, NY, USA) in 5% CO_2_ at 37 °C.

### 4.6. Assay of Osteoblast Proliferation and ALP Activity

MC3T3-E1 cells were plated and cultured for 24 h in 96-well plates at a density of 5 × 10^3^ cells/well for proliferation assay, or 24-well plates at a density of 2 × 10^4^ cells/well for ALP activity assay, and then treated with CAT, ACT, or ECH at concentrations of 10^−8^, 10^−7^, 10^−6^ M for 48 h, and then cultured in α-MEM or high level of glucose (200 mM) α-MEM for another 48 h. As for proliferation assay, 100 μL fresh medium containing 10 μL CCK-8 solution was added to each well, and the cells were incubated at 37 °C for 1 h. The absorbance at 450 nm was measured using a microplate reader. For ALP activity assay, cells were gently washed twice with phosphate-buffered saline and then lysed with 0.2% Triton X-100. The ALP activity and protein concentration in the lysate were determined using an ALP activity assay kit and a BCA-protein assay kit, respectively. The ALP activity was expressed as micromoles of p-nitrophenol liberated per-nanogram protein.

The regulatory effects of CAT, ACT, or ECH on BMP and IGF-1/PI3K/mTOR pathways of MC3T3-E1 cells were verified by adding the inhibitors of pathways (200 ng/mL noggin and 2 × 10^−7^ M picropodophyllin) in cultured cells, and the proliferation and ALP activity were measured as previously described.

### 4.7. Measurement of IGF-1 Level in Osteoblasts

MC3T3-E1 cells were incubated in 48-well plates at a density of 1 × 10^4^ cells/well for 24 h, and then treated with CAT, ACT, or ECH at a concentration of 10^−8^ and 10^−7^ M for 4 h. The medium was harvested for detection of IGF-1 level using ELISA kits.

### 4.8. Western Blotting

MC3T3-E1 cells were plated in 6-well plates at a density of 2 × 10^5^ cells/well for 24 h, treated with CAT, ACT, or ECH at a concentration of 10^−8^ and 10^−7^ M for 4 h. After removing the medium, cells were lysed in a buffer (P0013 and 1 mM PMSF, Beyotime Institute of Biotechnology, Shanghai, China) for 30 min at 4 °C. The supernatant was harvested by centrifuging at 12,000 *g* for 5 min at 4 °C. A BCA Kit was used to measure protein concentrations. The proteins were separated by 10% SDS-PAGE and transferred to nitrocellulose membranes. The membranes were blocked with 5% BSA for 1 h, and then detected for anti-IGF-1R, anti-p-IGF-1R, anti-BMP2, anti-Runx2, anti-Osterix, anti-AKT, anti-p-AKT, anti-PI3K, anti-p-PI3K (Abcam, Cambridge, UK), anti-p-Smad1/5/9, anti-p-mTOR, anti-mTOR, anti-β-actin (Cell Signaling Technology, Beverly, MA, USA), and anti-Smad1 (Boster, Wuhan, China) at 4 °C overnight, and then incubated with anti-goat or anti-rabbit IgG conjugated with horseradish peroxidase (HRP) for 1 h at room temperature. The Infrared Imaging System (LI-COR Biosciences, Lincoln, NE, USA) was used to scan and analyze the images.

### 4.9. Molecular Docking

The IGF-1 receptor structures were prepared with the Protein Preparation Wizard Workflow provided in the Maestro module of Schrödinger software (Schrödinger, LLC: New York, NY, USA, 2017). PDB ID 5HZN was selected. The protein was processed by the default pipeline, including deleting water molecules, adding missing side chains and hydrogen atoms, creating a zero-order bond to metals, assigning protonation states and partial charges, and a restrained minimization with the root-mean-square deviation (RMSD) for hydrogen atoms reached of 0.3 Å. Subsequently, the receptor grids of these complexes were generated with the Glide module of Schrödinger software, and the grid boxes were defined as a 12 × 12 × 12 Å, with the space region centered at the original ligand of the complex structures. The 3D structures of compounds (CAT, ACT, and ECH) as well their deglycosylation products were built by Chem3D Ultra 8.0 and LigPrep (Schrödinger, LLC: New York, NY, USA, 2017) was applied to generate stereoisomers and tautomers, and the protonation states of ligands at pH 7.0 ± 2.0 were predicted by Epik (Schrödinger, LLC: New York, NY, USA, 2017). Other parameters were set as default. Ligand docking module in Schrödinger was used to choose the receptor grid file and ligands file, which generated from ligand preparation and protein preparation. Settings used SP (standard precision) or XP (extra precision), and the output writes out at most 3 poses per ligand. For other parameters, the default values were assigned. The binding mode was visualized by Pymol [48].

### 4.10. Statistical Analysis

Statistical analyses were performed with Prism 7.0 (GraphPad, San Diego, CA, USA). All data are expressed as means ± standard deviation (SD). Statistical comparisons were performed by using the Student’s t-test between two groups or one-way ANOVA for multiple groups followed by Fisher’s least significant difference test. The difference was considered statistically significant when *p* < 0.05.

## 5. Conclusions

The present study showed that RR prevented bone loss in diabetic rats. CAT, ACT, and ECH from RR increased the proliferation and differentiation of osteoblastic MC3T3-E1 cells injured by high glucose and promoted the production of IGF-1 and expression of related proteins in BMP and IGF-1/PI3K/mTOR signaling pathways. The verifying test of the inhibitor of IGF-1/PI3K/mTOR pathways (picropodophyllin) and molecular docking of IGF-1R further indicated that CAT, ACT, and ECH extracted from RR enhanced bone formation by regulating IGF-1/PI3K/mTOR signaling pathways, which may to some extent explain the mechanism of RR in preventing diabetic osteoporosis. Consequently, our findings suggest that RR could be regarded as a potential candidate drug for the treatment of diabetic osteoporosis.

## Supplementary Materials

Supplementary materials can be found at https://www.mdpi.com/1422-0067/20/16/3964/s1.

## Abbreviations

ACT	acteoside
AKT	protein kinase B
ALP	alkaline phosphatase
BMC	bone mineral content
BMD	bone mineral density
BMP	bone morphogenic protein
Calib.Tb.Th.3D	three-dimensional calibration of trabecular thickness
CAT	catalpol
DM	diabetes mellitus
DOP	diabetic osteoporosis
DPD	deoxypyridinoline
ECH	echinacoside
IGF-1	insulin-like growth factor-1
IGF-1R	insulin-like growth factor-1 recepter
MAPK	mitogen-activated protein kinase
Micro-CT	micro-computed tomography
mTOR	mammalian target of rapamycin complex 1
RANKL	receptor activator of nuclear factor kappa-Β ligand
NF-κB	nuclear factor kappa-Β
NFATc1	Nuclear factor of activated T-cells, cytoplasmic 1
NOG	noggin
OCN	osteocalcin
OVX	ovariectomized
PI3K	phosphatidylinositide 3-kinases
PPP	picropodophyllin
RR	Rhmanniapraeparata Radix
SMI	structure model index
STZ	streptozotocin
Tb.N	trabecular number
Tb.Th	trabecular thickness
Tb.Sp	trabecular separation
TRAP	tartrate resistant acid phosphatase
