# Supplementary figures and images for: Rehmannia glutinosa Libosch Extracts Prevent Bone Loss and Architectural Deterioration and Enhance Osteoblastic Bone Formation by Regulating the IGF-1/PI3K/mTOR Pathway in Streptozotocin-Induced Diabetic Rats
Source: Int J Mol Sci. 2019 Aug 15;20(16):3964. doi: 10.3390/ijms20163964 (PMC6720794; doi:10.3390/ijms20163964)

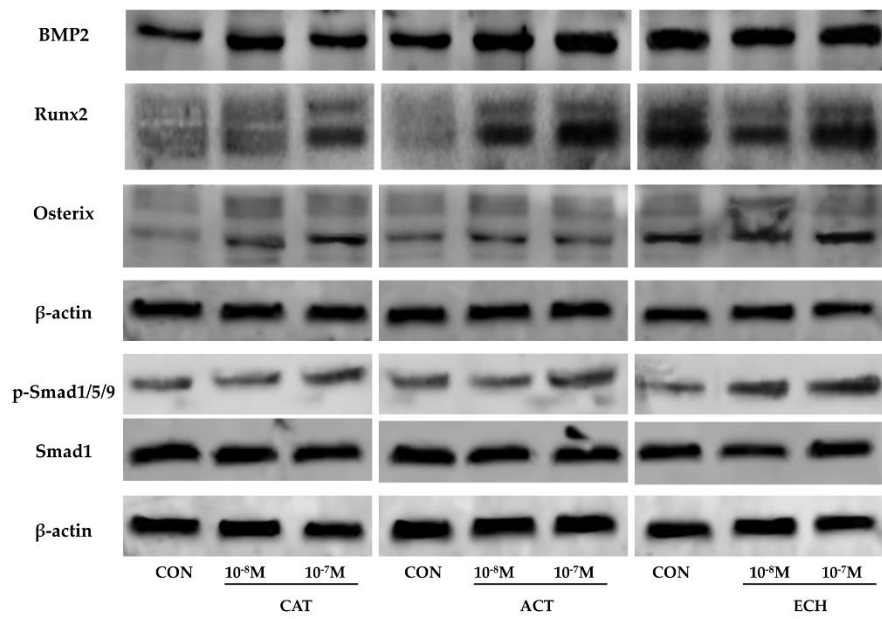

Figure S1

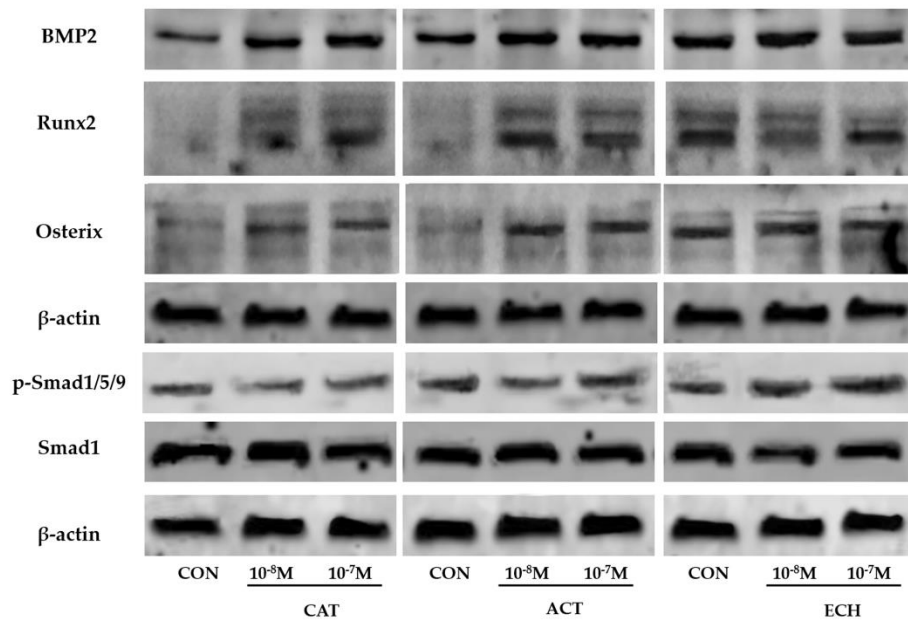

Figure S2

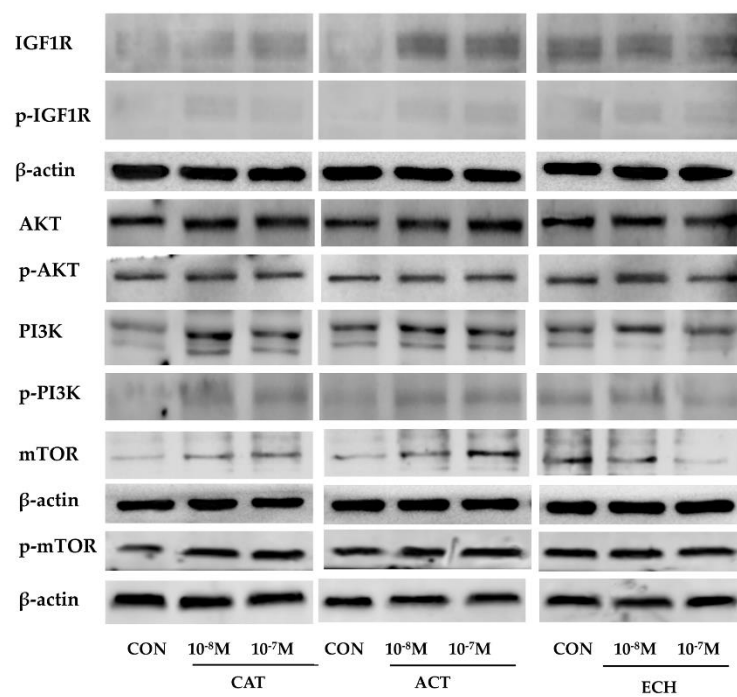

Figure S3

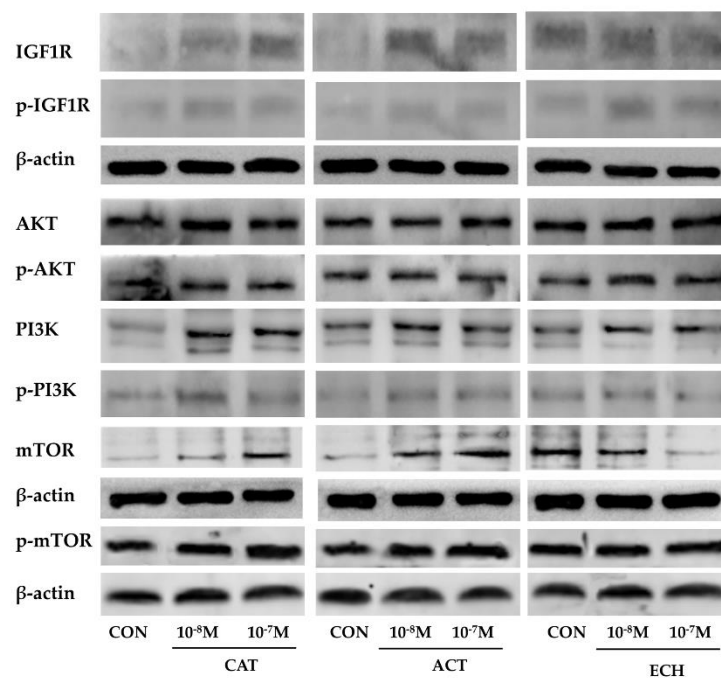

Figure S4

Supplement: Supplementary file 1 [file ijms-20-03964-s001.pdf]
